# The Notification of Ophthalmia Neonatorum in Scotland

**Published:** 1918-09-14

**Authors:** 


### The Notification of Ophthalmia Neonatorum in Scotland.

The Scottish Local Government Board has just issued an Order providing for the compulsory notification of ophthalmia neonatorum in Scotland after November 1, 1918. The Order applies not only to registered medical practitioners, but to every certified midwife and to every woman other than a certified midwife attending a child in the capacity of a nurse. Ophthalmia neonatorum is defined as any inflammation occurring in the eyes of an infant within twenty-one days from the date of its birth, and accompanied by a discharge. Notification is to be made by a medical practitioner when he first becomes aware that a child upon whom he is in professional attendance is suffering from the disease, and by a midwife or nurse as soon as she has reasonable grounds for supposing that a child upon whom she is in attendance in the course of her practice as a midwife or in the capacity of a nurse is so suffering. A fee of half a crown will be paid to a medical practitioner for each notification if the case occurs in his private practice, and of one shilling if the case occurs in his practice as medical officer of a public body or institution. The fee payable to a midwife or nurse will be one shilling, and so long as Section 5 of the Local Government (Emergency Provisions) Act, 1916, remains in force the fee payable to all medical practitioners will be limited to the same amount.

In the letter accompanying the Order the Scottish Local Government Board points out that mere notification will neither prevent nor cure the disease, and that it is imperative that skilled assistance should be provided for all cases notified. The treatment recommended is frequent cleansing of the eyes and the application at regular intervals of strong germicides. The Board considers that the cleansing of the eyes can be done by a trained nurse or, if no trained nurse is available, by a competent woman under instruction or after instruction, but that strong germicides should not be used except under the supervision or special direction of a medical man. The child must be visited every day by a trained nurse or by a medical man to see what progress is being made. The Board is of opinion that the treatment can be carried out in most cases in the child's home, but that in a certain number of cases it is essential to provide hospital accommodation for the child and, if necessary, for the mother also.

These recommendations are open to one very grave objection. The course of the disease is often so rapid that great skill and experience and energetic treatment may at any moment be required to avert calamity. Every case of ophthalmia neonatorum should be under the close supervision of a medical man. One is surprised that the Scottish Local Government Board should suggest that any case can be safely treated by an untrained woman, supplemented by a daily visit from a trained nurse.

